# Bis{bis­[1-meth­oxy-2-(2-meth­oxy­eth­oxy)ethane-κ^3^ *O*,*O*′,*O*′′]sodium} 1,1,2,2-tetra­phenyl­ethane-1,2-diide

**DOI:** 10.1107/S1600536814012823

**Published:** 2014-06-07

**Authors:** Mikhail E. Minyaev, John E. Ellis

**Affiliations:** a A.V. Topchiev Institute of Petrochemical Synthesis, Russian Academy of Sciences, 29 Leninsky Prospect, 119991 Moscow, Russian Federation; b University of Minnesota, 207 Pleasant St. SE, Minneapolis, MN 55455, USA

## Abstract

Crystals of the title salt, [Na(C_6_H_14_O_3_)_2_]_2_(C_26_H_20_), were grown from a tetra­hydro­furan/diglyme/Et_2_O solvent mixture [diglyme is 1-meth­oxy-2-(2-meth­oxy­eth­oxy)ethane]. The cations and dianion are separated in the crystal structure, unlike in the other three structurally characterized dialkali metal tetra­phenyl­ethyl­ene salts. The asymmetric unit contains one [Na(diglyme)_2_]^+^ cation and one half of the [Ph_2_CCPh_2_]^2−^ dianion. The latter lies on a twofold rotation axis. C—C bond-length redistribution displays that excessive electron density of the dianion is predominantly located at the C atoms of a former double bond and at all eight *ortho* positions. The studied crystal was a twin, with the ratio of two major components being 0.2143 (9):0.7857 (9). The twin operation is a twofold rotation around the *a* axis.

## Related literature   

For the crystal structure of free tetra­phenyl­ethyl­enide, see: Hua *et al.* (2007 ▶). For the preparation and reactivity of disodiumtetra­phenyl­ethyl­ene, see: Schlenk & Bergmann (1928 ▶). For UV–VIS data, see: Roberts & Szwarc (1965 ▶). For ^1^H and ^13^C{^1^H} NMR spectra, see: Roitershtein *et al.* (1998 ▶). For crystal structures of related alkali-metal tetra­phenyl­ethyl­ene salts, see: Bock *et al.* (1989 ▶, 1996 ▶); Minyaev *et al.* (2007 ▶). For crystal structures of hetero- and homoleptic *d-* and *f-*metal complexes with the tetra­phenyl­ethilene dianion, see: Roitershtein *et al.* (1998 ▶, 2004 ▶, 2007 ▶); Minyaev *et al.* (2007 ▶).
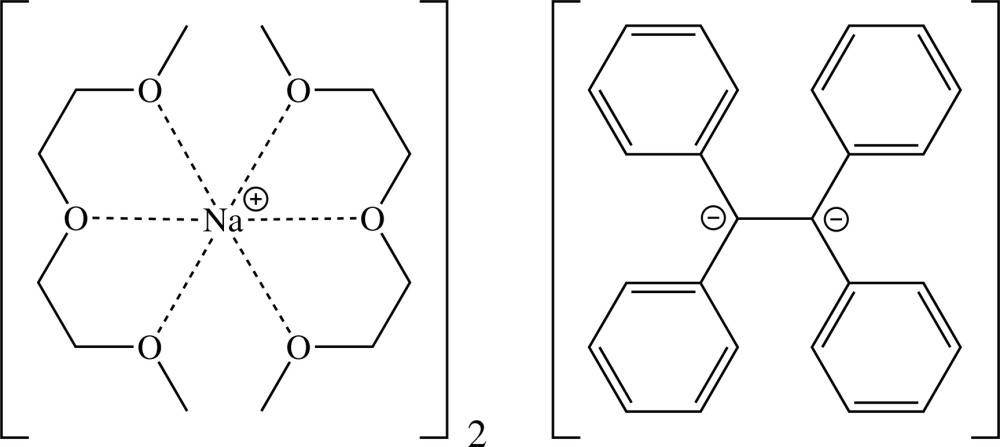


## Experimental   

### 

#### Crystal data  

[Na(C_6_H_14_O_3_)_2_]_2_(C_26_H_20_)
*M* *_r_* = 915.08
Monoclinic, 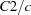

*a* = 10.048 (2) Å
*b* = 24.165 (5) Å
*c* = 20.978 (4) Å
β = 92.92 (3)°
*V* = 5087.0 (18) Å^3^
*Z* = 4
Mo *K*α radiation
μ = 0.10 mm^−1^
*T* = 123 K
0.50 × 0.30 × 0.20 mm

#### Data collection  

Siemens SMART Platform CCD diffractometer
Absorption correction: multi-scan (*TWINABS*; Bruker, 2003 ▶) *T*_min_ = 0.657, *T*_max_ = 0.746
6286 measured reflections
6286 independent reflections
5347 reflections with *I* > 2σ(*I*)

#### Refinement  

*R*[*F* ^2^ > 2σ(*F* ^2^)] = 0.048
*wR*(*F* ^2^) = 0.115
*S* = 1.06
6286 reflections
294 parameters
H-atom parameters constrained
Δρ_max_ = 0.42 e Å^−3^
Δρ_min_ = −0.23 e Å^−3^

### 

Data collection: *SMART* (Bruker, 2003 ▶); cell refinement: *CELL_NOW* (Sheldrick, 2003 ▶) and *SAINT* (Bruker, 2003 ▶); data reduction: *SAINT*; program(s) used to solve structure: *SHELXS2012* (Sheldrick, 2008 ▶); program(s) used to refine structure: *SHELXL2012* (Sheldrick, 2008 ▶); molecular graphics: *SHELXTL* (Sheldrick, 2008 ▶); software used to prepare material for publication: *SHELXTL2012* (Sheldrick, 2008 ▶) and *publCIF* (Westrip, 2010 ▶).

## Supplementary Material

Crystal structure: contains datablock(s) I, New_Global_Publ_Block. DOI: 10.1107/S1600536814012823/pj2012sup1.cif

Structure factors: contains datablock(s) I. DOI: 10.1107/S1600536814012823/pj2012Isup2.hkl

Supporting information file. DOI: 10.1107/S1600536814012823/pj2012Isup3.cdx

CCDC reference: 1006312

Additional supporting information:  crystallographic information; 3D view; checkCIF report

## Figures and Tables

**Table 1:** Selected bond lengths (Å)

C1—C1^i^	1.507 (3)
C1—C2	1.438 (2)
C1—C8	1.429 (2)
C2—C3	1.424 (3)
C2—C7	1.434 (3)
C3—C4	1.384 (3)
C4—C5	1.390 (3)
C5—C6	1.390 (3)
C6—C7	1.387 (3)
C8—C9	1.433 (3)
C8—C13	1.436 (3)
C9—C10	1.380 (3)
C10—C11	1.389 (3)
C11—C12	1.394 (3)
C12—C13	1.378 (3)

Symmetry code: (i) 
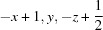
.

**Fig. 1.**
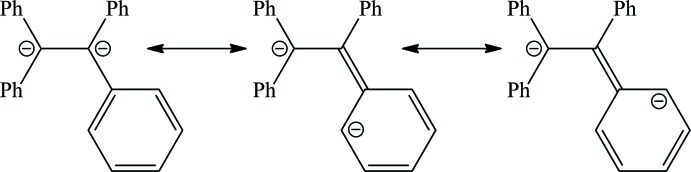
Some of possible resonance forms, showing charge redistribution among one C= and two Cortho positions.

**Fig. 2.**
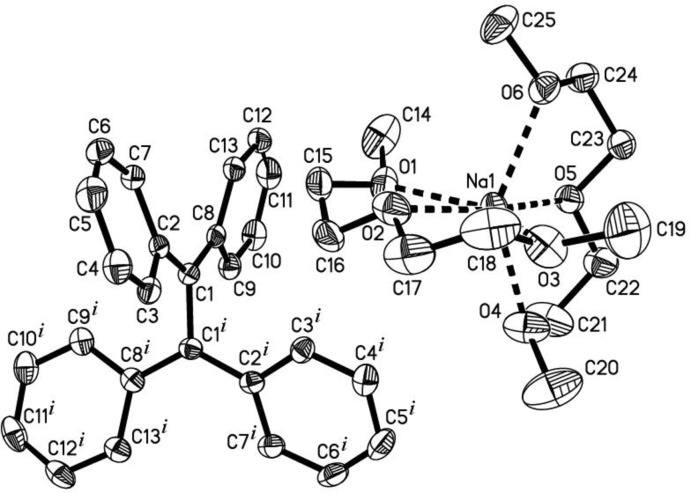
Molecular structure of [Na(diglyme)2]+2[Ph2CCPh2]2- (50% atomic displacement parameters). One cation is shown. Hydrogen atoms are omitted for clarity. Symmetry code: (i) -x + 1, y, -z + 1/2.

## supplementary crystallographic information

### S1. Comment

Detailed preparation and reactivity of disodiumtetraphenylethylene were described by Schlenk & Bergmann (1928). Since then, properties of the tetraphenylethylene dianion and radical-anion have been intensively studied, including electronic spectra, ^1^H and ^13^C NMR spectral data, electrochemistry and *etc*. For example, ligand to metal charge transfer absorption band of the disodiumtetraphenylethylene in THF solution was reported by Roberts & Szwarc (1965); ^1^H and ^13^C{^1^H} NMR spectra of disodiumtetraphenylethylene in THF_d-8_ have been reported by Roitershtein *et al.* (1998).

Synthetic applications of the tetraphenylethylene dianion are remarkably few. Reactivity of the tetraphenylethylene dianion towards formation either d- or f-element complexes is not well established so far. Only five crystal structures of hetero- and homoleptic complexes of lanthanides and yttrium with the tetraphenylethylene dianion have been reported: [Na(thf)_6_]_2_[Y(Ph_4_C_2_)_2_](thf)_2_, (Roitershtein *et al.*, 1998), [Na(diglyme)_2_][Lu(Ph_4_C_2_)_2_](thf)_0.5_ (Roitershtein *et al.*, 2004), [Na(thf)_3_][Na(thf)_4_][Yb(Ph_4_C_2_)_2_] (Minyaev *et al.*, 2007), [(C_5_H_5_)Lu(Ph_4_C_2_)(dme)](dme) (Roitershtein *et al.*, 2004) (dme is 1,2-dimethoxyethane), [(1,3–Ph_2_C_5_H_3_)Lu(Ph_4_C_2_)(thf)] (Roitershtein *et al.*, 2007).

As a part of our research project on synthesis and structural characterization of new tetraphenylethenide derivatives of rare-earth metals, we have explored reaction of disodiumtetraphenylethylene with anhydrous scandium(III) iodide (2:1 molar ratio), which was supposed to lead to formation of a homoleptic tetraphenylethenide *ate*-complex Na[Sc(Ph_4_C_2_)_2_]. Anhydrous ScI_3_ was activated by refluxing in THF in order to destroy its polymeric structure. According to ^1^H NMR and UV-VIS spectral data, the final product after work-up contained expected Na[Sc(Ph_4_C_2_)_2_] and some unreacted Na_2_[Ph_4_C_2_]. Most likely, ScI_3_ coordination polymer was not fully converted into the monomer or short oligomers, therefore, the tetraphenylethylene dianion was not fully consumed. Attempts to grow crystals of the reaction product in various solvents and conditions allowed us to obtain only crystals of the title compound when diglyme was used as σ-donating chelating ligand. Analogous reactions of *M*_2_[Ph_4_C_2_] (*M*= Na, K) with LnCl_3_(thf)_3_ (Ln=Sc, Y, Lu), which have a molecular structure, go smoothly without any complications.

Crystal structure of free tetraphenylethylene is well known (see, for example, Hua *et al.*, 2007). However, only three X-ray crystal structures of solvated dialkali metal tetraphenylethenides have been known up to date: {Na[Na(Ph_4_C_2_)(Et_2_O)_2_]}_n_ (Bock *et al.*, 1989); {[Cs(diglyme)]_2_[Ph_4_C_2_]} (Bock *et al.*, 1996); {[K(dme)_2_]_2_[Ph_4_C_2_]} (Minyaev *et al.*, 2007). There are interionic short contacts *M*^+^–Ph_4_C_2_^2-^ (*M*= Na, K, Cs) in all three crystal structures, which may have some impact on the C—C bond length redistribution inside the easily polarizable tetraphenylethylene dianion.

The title compound, [Na(diglyme)_2_]^+^_2_[Ph_2_CCPh_2_]^2-^, is the only example with separated counterions in crystalline lattice. Consequently, one may suppose that the C—C bond distances of the dianion are relatively unperturbed. However, some non-covalent interionic bonding is present. A three-dimensional framework is formed by C—H_diglyme_···C_Ph_π-interactions (*e.g.*, C14—H14A···C13, C15—H15A···C2, C17—H17B···C11, C22—H22A···η^2^-C10—C11, C23—H23A···η^6^-C2—C3—C4—C5—C6—C7) and by C—H_Ph_···O_diglyme_ hydrogen bonds (C4—H4···O4). The asymmetric unit contains one cation [Na(diglyme)_2_]^+^ and a half of the dianion [Ph_2_CCPh_2_]^2-^. The center of the tetraphenylethylene former C–C double bond (C_=_–C_=_) lies on a 2-fold rotation axis. The dianion accommodates a twist conformation. The angle between two planes, formed by C*_ipso_*, C_=_ and C*_ipso_* carbon atoms (C2—C1—C8 and C2^i^—C1^i^—C8^i^, respectively), equals to 82.4°. The C_=_–C_=_ bond (atoms C1–C1^i^) is elongated up to a single bond (1.507 (3) Å, see table 1). Based on table 1, the C_=_–C*_ipso_* bond lengths are shorter and C*_ipso_*–C*_ortho_* — longer than those in free tetraphenylethylene. Averaged C—C bond distances inside the dianion are 1.434 Å (C_=_–C*_ipso_*), 1.432 Å (C*_ipso_*–C*_ortho_*), 1.382 Å (C*_ortho_*–C*_meta_*), 1.391 Å (C*_meta_*–C*_para_*). For comparison, the same C—C bond distances in free tetraphenylethylene (Hua *et al.*, 2007) are 1.356 (2) Å (C_=_–C_=_), 1.494 Å (C_=_–C*_ipso_*), 1.397 Å (C*_ipso_*–C*_ortho_*), 1.388 Å (C*_ortho_*–C*_meta_*), 1.384 Å (C*_meta_*–C*_para_*).

The observed C—C bond length redistribution inside the dianion could be rationalized by localization of excessive negative charge predominantly not only at C_=_ carbon atoms but also at all eight C*_ortho_* positions (see Fig.1).

Similar averaged C—C bond lengths in the Ph_4_C_2_^2-^ dianion can be found in {[Cs(diglyme)]_2_[Ph_4_C_2_]} (Bock *et al.*, 1996) — 1.51 (1) Å (C_=_–C_=_), 1.436 Å (C_=_–C*_ipso_*), 1.423 Å (C*_ipso_*–C*_ortho_*), 1.387 Å (C*_ortho_*–C*_meta_*), 1.385 Å (C*_meta_*–C*_para_*), and in {[K(dme)_2_]_2_[Ph_4_C_2_]} (Minyaev *et al.*, 2007) — 1.496 (2) Å (C_=_–C_=_), 1.433 Å (C_=_–C*_ipso_*), 1.430 Å (C*_ipso_*–C*_ortho_*), 1.384 Å (C*_ortho_*–C*_meta_*), 1.392 Å (C*_meta_*–C*_para_*). Therefore, M—Ph_4_C_2_ (*M*=K, Cs) short contacts do not introduce much influence on the C—C bond length redistribution, compared to that of the title compound with separated counterions.

In coordination polymer {Na[Na(Ph_4_C_2_)(Et_2_O)_2_]}_n_ (Bock *et al.*, 1989), the C_=_–C_=_ bond length is 1.487 Å, but some similar C—C distances vary essentially. Thus, phenyl rings coordinated with Na^+^ and Na(Et_2_O)_2_^+^ moieties display the following averaged bond lengths: 1.463 Å (C_=_–C*_ipso_*), 1.444 Å (C*_ipso_*–C*_ortho_*), 1.397 Å (C*_ortho_*–C*_meta_*), 1.396 Å (C*_meta_*–C*_para_*). For phenyl rings that almost do not interact with the Na^+^ cation, the averaged C—C bond distances are 1.436 Å (C_=_–C*_ipso_*), 1.416 Å (C*_ipso_*–C*_ortho_*. Actual bond lengths are 1.407 Å, 1.423 Å for a one phenyl ring and 1.405 Å, 1.427 Å for another one.), 1.392 Å (C*_ortho_*–C*_meta_*), 1.391 Å (C*_meta_*–C*_para_*). It is obvious that excessive electron density of the dianion is not equally localized at *ortho*-positions of coordinated and uncoordinated phenyl groups, and charge redistribution inside the dianion is quite different from such of three previous salts. Impact on C—C bond distances could be partly explained by stronger polarizing ability of Na^+^, compared to K^+^ and Cs^+^.

It is worth mentioning that the angle between two C*_ipso_*—C_=_—C*_ipso_* planes decreases in the following order: [Na(diglyme)_2_]_2_[Ph_2_CCPh_2_] (82.4°), {[Cs(diglyme)]_2_[Ph_4_C_2_]} (75.5°), {[K(dme)_2_]_2_[Ph_4_C_2_]} (68.7°), {Na[Na(Ph_4_C_2_)(Et_2_O)_2_]}_n_ (56.1°), which has a positive correlation with the M—Ph_4_C_2_ strength interaction (*M*=Na, K, Cs). The same angle is 8.8° in free Ph_2_C=CPh_2_.

The C—C bond length redistribution changes even more dramatically in crystalline lattice of rare-earth complexes due to higher polarizing ability of Ln^3+^ and Yb^2+^. The coordinated tetraphenylethelene dianion in rare-earth complexes exhibits bis-η^3^-allylic coordination mode, where the excessive negative charge of the dianion is localized at C_=_ carbon atoms and only at two C*_ortho_* positions coordinated with the metal cation (Roitershtein *et al.*, 1998; Roitershtein *et al.*, 2004; Minyaev *et al.*, 2007; Roitershtein *et al.*, 2007).

### S2. Experimental

All synthetic manipulations were carried out under vacuum or argon atmosphere, using Schlenk glassware, dry box techniques and absolute solvents. A 250 ml flask with freshly cut sodium metal (0.60 g, 26 mmol) was evacuated. Sodium mirror was formed on the flask's walls upon heating under dynamic vacuum. Tetraphenylethylene solution in THF (1.249 g, 3.76 mmol in 100 ml) was added into the flask. The solution was stirred for 1 day, the color changed to purple (λ_max_= 485 nm). Simultaneously, anhydrous ScI_3_ (0.784 g, 1.84 mmol) was stirred for 5 h in THF (50 ml) and then refluxed for 2 h while stirring to form a finely divided suspension. The Na_2_[Ph_4_C_2_] solution was added dropwise into the ScI_3_ suspension. The reaction mixture was stirred for 1 day. λ_max_ of the reaction mixture = 422 nm (very br.), which is a superposition of 2 ligand to metal charge transfer absorption bands: 485 nm for Na_2_[Ph_4_C_2_] (Roberts & Szwarc, 1965) and 392 nm for Na[Sc(Ph_4_C_2_)_2_]. λ_max_ did not change after 3 day of stirring. Then the reaction mixture was filtered, the filter cake and the flask were washed with THF (2x20 ml). Most THF was evaporated from the solution, leaving some solid and c.a. 20 ml of the solution. Pentane (c.a. 100 ml) was layered on a top of the solution. After 1 day, the mixture was stirred. Precipitate was filtered off, washed with pentane (2x20 ml) and with diethyl ether (2x20 ml), dried under vacuum for 4 h. The yield of crude product was 1.473 g.

To grow crystals of the title compound, the obtained crude product (150 mg) was dissolved in mixture of THF (20 ml) and diglyme (5 ml). Diethyl ether (35 ml) was layered on a top of the resulting solution. After 2 weeks, crystals formed on Schlenk tube's walls.

The ^1^H NMR spectrum of the crude product consisted of two sets of proton signals. The first set belongs to Na_2_[Ph_4_C_2_] (see Roitershtein *et al.*, 1998). The second set corresponds to Na[Sc(Ph_4_C_2_)_2_] – ^1^H NMR (300 MHz, -30°C, THF_d-8_, δ, p.p.m.): 4.65 (dd, 4H), 6.29 (t, 4H), 6.58 (t, 4H), 6.87 (br.t, 12H), 7.07 (t, 4H), 7.14 (d, 8H), 7.65 (dd, 4H).

Based on qualitative analysis with xylenol orange indicator, the filter cake contained significant amounts of scandium.

### S3. Refinement

The hydrogen atoms were positioned geometrically (C—H distance = 0.95 Å for aromatic, 0.98 Å for methyl, 0.99 Å for methylene protons) and refined as riding atoms with *U*_iso_(H)= 1.5*U*_eq_(C) for methyl protons, 1.2*U*_eq_(C) for other protons. A rotating group model was applied for methyl groups. The studied crystal was a twin with a ratio of two major domains of 0.2143 (9): 0.7857 (9). The two domains were rotated from each other by 180.0° about a real axis (1 0 0), which was determined by Cell Now program (Sheldrick, 2003). The final refinement was carried out using detwinned data set.

Disorder of the C20—O4—C21—C22 cation fragment was not modeled, since the residual electron density was not sufficient to model the disorder properly.

### Crystal data

**Table:**

[Na(C_6_H_14_O_3_)_2_]_2_(C_26_H_20_)	*F*(000) = 1976
*M**_r_* = 915.08	*D*_x_ = 1.195 Mg m^−^^3^
Monoclinic, *C*2/*c*	Mo *K*α radiation, λ = 0.71073 Å
*a* = 10.048 (2) Å	Cell parameters from 4057 reflections
*b* = 24.165 (5) Å	θ = 2.2–28.2°
*c* = 20.978 (4) Å	µ = 0.10 mm^−^^1^
β = 92.92 (3)°	*T* = 123 K
*V* = 5087.0 (18) Å^3^	Block, red
*Z* = 4	0.50 × 0.30 × 0.20 mm

### Data collection

**Table:**

Siemens SMART Platform CCD diffractometer	6286 independent reflections
Radiation source: normal-focus sealed tube	5347 reflections with *I* > 2σ(*I*)
Graphite monochromator	*R*_int_ = 0.0
area detector, ω scans per phi	θ_max_ = 28.3°, θ_min_ = 1.7°
Absorption correction: multi-scan (*TWINABS*; Bruker, 2003)	*h* = −13→13
*T*_min_ = 0.657, *T*_max_ = 0.746	*k* = 0→32
6286 measured reflections	*l* = 0→27

### Refinement

**Table:**

Refinement on *F*^2^	Primary atom site location: structure-invariant direct methods
Least-squares matrix: full	Secondary atom site location: difference Fourier map
*R*[*F*^2^ > 2σ(*F*^2^)] = 0.048	Hydrogen site location: inferred from neighbouring sites
*wR*(*F*^2^) = 0.115	H-atom parameters constrained
*S* = 1.06	*w* = 1/[σ^2^(*F*_o_^2^) + (0.0435*P*)^2^ + 4.6083*P*] where *P* = (*F*_o_^2^ + 2*F*_c_^2^)/3
6286 reflections	(Δ/σ)_max_ < 0.001
294 parameters	Δρ_max_ = 0.42 e Å^−^^3^
0 restraints	Δρ_min_ = −0.23 e Å^−^^3^

### Special details

**Table:**

Experimental. moisture and air sensitive
Geometry. All e.s.d.'s (except the e.s.d. in the dihedral angle between two l.s. planes) are estimated using the full covariance matrix. The cell e.s.d.'s are taken into account individually in the estimation of e.s.d.'s in distances, angles and torsion angles; correlations between e.s.d.'s in cell parameters are only used when they are defined by crystal symmetry. An approximate (isotropic) treatment of cell e.s.d.'s is used for estimating e.s.d.'s involving l.s. planes.
Refinement. Refinement of *F*^2^ against ALL reflections. The weighted *R*-factor *wR* and goodness of fit *S* are based on *F*^2^, conventional *R*-factors *R* are based on *F*, with *F* set to zero for negative *F*^2^. The threshold expression of *F*^2^ > σ(*F*^2^) is used only for calculating *R*-factors(gt) *etc*. and is not relevant to the choice of reflections for refinement. *R*-factors based on *F*^2^ are statistically about twice as large as those based on *F*, and *R*- factors based on ALL data will be even larger. Refined as a 2-component twin. BASF=0.214

### Fractional atomic coordinates and isotropic or equivalent isotropic displacement parameters (Å^2^)

**Table:**

	*x*	*y*	*z*	*U*_iso_*/*U*_eq_	
C1	0.42755 (17)	0.13927 (7)	0.25769 (8)	0.0214 (3)	
C2	0.38922 (17)	0.17452 (7)	0.30886 (8)	0.0219 (3)	
C3	0.47563 (19)	0.21800 (7)	0.33076 (8)	0.0251 (4)	
H3	0.5576	0.2228	0.3108	0.030*	
C4	0.4459 (2)	0.25360 (8)	0.37973 (9)	0.0326 (4)	
H4	0.5062	0.2825	0.3915	0.039*	
C5	0.3292 (2)	0.24769 (9)	0.41196 (9)	0.0379 (5)	
H5	0.3084	0.2723	0.4453	0.045*	
C6	0.2442 (2)	0.20449 (9)	0.39376 (9)	0.0346 (4)	
H6	0.1654	0.1990	0.4162	0.042*	
C7	0.27099 (19)	0.16925 (8)	0.34386 (8)	0.0273 (4)	
H7	0.2091	0.1407	0.3325	0.033*	
C8	0.33820 (18)	0.10525 (7)	0.21972 (8)	0.0221 (3)	
C9	0.3903 (2)	0.06401 (7)	0.17867 (9)	0.0276 (4)	
H9	0.4842	0.0598	0.1779	0.033*	
C10	0.3100 (2)	0.03023 (8)	0.14016 (9)	0.0343 (4)	
H10	0.3500	0.0033	0.1142	0.041*	
C11	0.1720 (2)	0.03466 (9)	0.13839 (10)	0.0383 (5)	
H11	0.1170	0.0109	0.1124	0.046*	
C12	0.1172 (2)	0.07532 (9)	0.17626 (10)	0.0344 (5)	
H12	0.0232	0.0797	0.1751	0.041*	
C13	0.19548 (19)	0.10935 (8)	0.21528 (9)	0.0271 (4)	
H13	0.1535	0.1365	0.2401	0.032*	
Na1	0.19803 (8)	0.37669 (3)	0.10184 (3)	0.03106 (18)	
C14	0.0899 (3)	0.25199 (9)	0.14723 (13)	0.0545 (7)	
H14A	0.1255	0.2159	0.1610	0.082*	
H14B	0.0679	0.2512	0.1012	0.082*	
H14C	0.0093	0.2601	0.1700	0.082*	
O1	0.18706 (16)	0.29378 (6)	0.16114 (6)	0.0393 (4)	
C15	0.2176 (2)	0.29889 (11)	0.22790 (10)	0.0453 (6)	
H15A	0.2590	0.2644	0.2448	0.054*	
H15B	0.1352	0.3058	0.2506	0.054*	
C16	0.3109 (2)	0.34577 (12)	0.23745 (10)	0.0499 (6)	
H16A	0.3346	0.3508	0.2835	0.060*	
H16B	0.3936	0.3387	0.2151	0.060*	
O2	0.24528 (15)	0.39425 (7)	0.21202 (7)	0.0409 (4)	
C17	0.3210 (3)	0.44350 (13)	0.21969 (12)	0.0576 (7)	
H17A	0.4123	0.4375	0.2055	0.069*	
H17B	0.3271	0.4546	0.2652	0.069*	
C18	0.2538 (3)	0.48737 (10)	0.18068 (13)	0.0573 (7)	
H18A	0.1606	0.4918	0.1930	0.069*	
H18B	0.3006	0.5231	0.1876	0.069*	
O3	0.25612 (17)	0.47153 (6)	0.11525 (8)	0.0458 (4)	
C19	0.2039 (4)	0.51249 (11)	0.07319 (17)	0.0764 (9)	
H19A	0.2505	0.5475	0.0817	0.115*	
H19B	0.1086	0.5173	0.0796	0.115*	
H19C	0.2161	0.5011	0.0290	0.115*	
C20	0.4897 (3)	0.40361 (14)	0.03195 (17)	0.0767 (10)	
H20A	0.5734	0.3834	0.0282	0.115*	
H20B	0.4985	0.4292	0.0681	0.115*	
H20C	0.4692	0.4245	−0.0074	0.115*	
O4	0.38983 (16)	0.36721 (7)	0.04155 (8)	0.0476 (4)	
C21	0.3632 (3)	0.32886 (12)	−0.00971 (13)	0.0573 (7)	
H21A	0.3594	0.2908	0.0076	0.069*	
H21B	0.4365	0.3303	−0.0395	0.069*	
C22	0.2354 (2)	0.34211 (10)	−0.04470 (10)	0.0403 (5)	
H22A	0.2401	0.3792	−0.0646	0.048*	
H22B	0.2160	0.3145	−0.0788	0.048*	
O5	0.13343 (14)	0.34110 (6)	0.00064 (6)	0.0321 (3)	
C23	0.01609 (19)	0.36844 (9)	−0.02316 (9)	0.0316 (4)	
H23A	−0.0179	0.3510	−0.0635	0.038*	
H23B	0.0353	0.4079	−0.0317	0.038*	
C24	−0.0849 (2)	0.36364 (10)	0.02655 (10)	0.0368 (5)	
H24A	−0.1696	0.3812	0.0111	0.044*	
H24B	−0.1026	0.3242	0.0355	0.044*	
O6	−0.03470 (15)	0.39049 (6)	0.08320 (7)	0.0360 (3)	
C25	−0.1223 (3)	0.38392 (10)	0.13393 (11)	0.0484 (6)	
H25A	−0.2067	0.4028	0.1230	0.073*	
H25B	−0.0813	0.4000	0.1731	0.073*	
H25C	−0.1390	0.3444	0.1407	0.073*	

### Atomic displacement parameters (Å^2^)

**Table:**

	*U*^11^	*U*^22^	*U*^33^	*U*^12^	*U*^13^	*U*^23^
C1	0.0197 (8)	0.0234 (8)	0.0211 (8)	0.0002 (7)	0.0015 (6)	0.0031 (6)
C2	0.0211 (8)	0.0239 (8)	0.0205 (8)	0.0045 (7)	0.0005 (6)	0.0062 (6)
C3	0.0286 (9)	0.0264 (9)	0.0203 (8)	0.0018 (7)	0.0025 (7)	0.0014 (7)
C4	0.0408 (11)	0.0292 (10)	0.0277 (9)	0.0020 (8)	−0.0006 (8)	−0.0026 (8)
C5	0.0461 (13)	0.0394 (11)	0.0286 (10)	0.0145 (10)	0.0071 (9)	−0.0055 (8)
C6	0.0293 (10)	0.0475 (12)	0.0279 (9)	0.0123 (9)	0.0088 (8)	0.0050 (8)
C7	0.0239 (9)	0.0338 (9)	0.0242 (8)	0.0032 (7)	0.0025 (7)	0.0055 (7)
C8	0.0246 (9)	0.0210 (8)	0.0207 (8)	−0.0023 (7)	−0.0001 (7)	0.0072 (6)
C9	0.0327 (10)	0.0241 (9)	0.0257 (9)	−0.0016 (7)	−0.0008 (7)	0.0023 (7)
C10	0.0478 (12)	0.0259 (9)	0.0287 (9)	−0.0052 (9)	−0.0019 (9)	−0.0007 (7)
C11	0.0459 (13)	0.0334 (10)	0.0344 (10)	−0.0174 (9)	−0.0115 (9)	0.0032 (8)
C12	0.0259 (10)	0.0419 (11)	0.0346 (10)	−0.0100 (9)	−0.0060 (8)	0.0123 (9)
C13	0.0257 (9)	0.0286 (9)	0.0266 (9)	−0.0013 (7)	−0.0006 (7)	0.0073 (7)
Na1	0.0402 (4)	0.0287 (4)	0.0236 (3)	0.0006 (3)	−0.0053 (3)	−0.0037 (3)
C14	0.086 (2)	0.0275 (11)	0.0514 (14)	−0.0008 (12)	0.0215 (14)	−0.0041 (10)
O1	0.0509 (9)	0.0384 (8)	0.0292 (7)	0.0107 (7)	0.0063 (7)	0.0061 (6)
C15	0.0422 (13)	0.0667 (15)	0.0274 (10)	0.0218 (12)	0.0066 (9)	0.0130 (10)
C16	0.0314 (12)	0.0934 (19)	0.0245 (10)	0.0162 (12)	−0.0013 (8)	0.0027 (11)
O2	0.0330 (8)	0.0578 (9)	0.0316 (7)	0.0016 (7)	−0.0030 (6)	−0.0143 (7)
C17	0.0473 (14)	0.0851 (19)	0.0401 (12)	−0.0176 (14)	−0.0006 (11)	−0.0301 (13)
C18	0.0553 (15)	0.0454 (13)	0.0728 (17)	−0.0110 (12)	0.0181 (14)	−0.0372 (13)
O3	0.0510 (10)	0.0311 (7)	0.0546 (9)	0.0033 (7)	−0.0039 (8)	−0.0116 (7)
C19	0.093 (2)	0.0369 (14)	0.099 (2)	0.0093 (15)	0.008 (2)	0.0144 (15)
C20	0.075 (2)	0.0665 (19)	0.092 (2)	−0.0355 (16)	0.0292 (18)	−0.0306 (17)
O4	0.0354 (8)	0.0539 (10)	0.0535 (10)	−0.0069 (7)	0.0021 (7)	−0.0270 (8)
C21	0.0390 (13)	0.0708 (18)	0.0615 (16)	0.0057 (12)	−0.0025 (12)	−0.0355 (14)
C22	0.0356 (12)	0.0547 (13)	0.0308 (10)	0.0024 (10)	0.0043 (8)	−0.0084 (9)
O5	0.0291 (7)	0.0430 (8)	0.0240 (6)	0.0017 (6)	0.0002 (5)	−0.0018 (6)
C23	0.0302 (10)	0.0405 (11)	0.0237 (9)	−0.0031 (8)	−0.0026 (7)	0.0000 (8)
C24	0.0314 (11)	0.0467 (12)	0.0325 (10)	−0.0039 (9)	0.0025 (8)	−0.0009 (9)
O6	0.0396 (8)	0.0414 (8)	0.0277 (7)	0.0008 (6)	0.0075 (6)	−0.0005 (6)
C25	0.0632 (16)	0.0432 (12)	0.0411 (12)	0.0041 (11)	0.0258 (12)	0.0012 (10)

### Geometric parameters (Å, º)

**Table:**

C1—C1^i^	1.507 (3)	C15—H15A	0.9900
C1—C2	1.438 (2)	C15—H15B	0.9900
C1—C8	1.429 (2)	C16—O2	1.434 (3)
C2—C3	1.424 (3)	C16—H16A	0.9900
C2—C7	1.434 (3)	C16—H16B	0.9900
C3—C4	1.384 (3)	O2—C17	1.417 (3)
C3—H3	0.9500	C17—C18	1.481 (4)
C4—C5	1.390 (3)	C17—H17A	0.9900
C4—H4	0.9500	C17—H17B	0.9900
C5—C6	1.390 (3)	C18—O3	1.426 (3)
C5—H5	0.9500	C18—H18A	0.9900
C6—C7	1.387 (3)	C18—H18B	0.9900
C6—H6	0.9500	O3—C19	1.410 (3)
C7—H7	0.9500	C19—H19A	0.9800
C8—C9	1.433 (3)	C19—H19B	0.9800
C8—C13	1.436 (3)	C19—H19C	0.9800
C9—C10	1.380 (3)	C20—O4	1.357 (3)
C9—H9	0.9500	C20—H20A	0.9800
C10—C11	1.389 (3)	C20—H20B	0.9800
C10—H10	0.9500	C20—H20C	0.9800
C11—C12	1.394 (3)	O4—C21	1.434 (3)
C11—H11	0.9500	C21—C22	1.481 (3)
C12—C13	1.378 (3)	C21—H21A	0.9900
C12—H12	0.9500	C21—H21B	0.9900
C13—H13	0.9500	C22—O5	1.434 (2)
Na1—O5	2.3510 (16)	C22—H22A	0.9900
Na1—O1	2.3639 (16)	C22—H22B	0.9900
Na1—O4	2.3694 (19)	O5—C23	1.420 (2)
Na1—O2	2.3742 (16)	C23—C24	1.496 (3)
Na1—O6	2.3747 (18)	C23—H23A	0.9900
Na1—O3	2.3781 (17)	C23—H23B	0.9900
Na1—C16	3.100 (2)	C24—O6	1.424 (3)
Na1—C23	3.126 (2)	C24—H24A	0.9900
C14—O1	1.425 (3)	C24—H24B	0.9900
C14—H14A	0.9800	O6—C25	1.424 (3)
C14—H14B	0.9800	C25—H25A	0.9800
C14—H14C	0.9800	C25—H25B	0.9800
O1—C15	1.424 (3)	C25—H25C	0.9800
C15—C16	1.478 (4)		
			
C8—C1—C2	124.90 (16)	O2—C16—Na1	47.30 (8)
C8—C1—C1^i^	117.83 (15)	C15—C16—Na1	81.98 (12)
C2—C1—C1^i^	117.26 (15)	O2—C16—H16A	110.2
C3—C2—C7	114.07 (16)	C15—C16—H16A	110.2
C3—C2—C1	119.72 (15)	Na1—C16—H16A	157.5
C7—C2—C1	126.15 (16)	O2—C16—H16B	110.2
C4—C3—C2	123.15 (17)	C15—C16—H16B	110.2
C4—C3—H3	118.4	Na1—C16—H16B	83.3
C2—C3—H3	118.4	H16A—C16—H16B	108.5
C3—C4—C5	121.09 (19)	C17—O2—C16	114.22 (19)
C3—C4—H4	119.5	C17—O2—Na1	109.88 (13)
C5—C4—H4	119.5	C16—O2—Na1	106.35 (12)
C4—C5—C6	117.74 (18)	O2—C17—C18	108.2 (2)
C4—C5—H5	121.1	O2—C17—H17A	110.1
C6—C5—H5	121.1	C18—C17—H17A	110.1
C7—C6—C5	121.88 (19)	O2—C17—H17B	110.1
C7—C6—H6	119.1	C18—C17—H17B	110.1
C5—C6—H6	119.1	H17A—C17—H17B	108.4
C6—C7—C2	122.00 (18)	O3—C18—C17	108.06 (19)
C6—C7—H7	119.0	O3—C18—H18A	110.1
C2—C7—H7	119.0	C17—C18—H18A	110.1
C1—C8—C9	119.78 (16)	O3—C18—H18B	110.1
C1—C8—C13	126.40 (17)	C17—C18—H18B	110.1
C9—C8—C13	113.75 (16)	H18A—C18—H18B	108.4
C10—C9—C8	122.88 (19)	C19—O3—C18	112.9 (2)
C10—C9—H9	118.6	C19—O3—Na1	121.42 (17)
C8—C9—H9	118.6	C18—O3—Na1	110.89 (14)
C9—C10—C11	121.5 (2)	O3—C19—H19A	109.5
C9—C10—H10	119.2	O3—C19—H19B	109.5
C11—C10—H10	119.2	H19A—C19—H19B	109.5
C10—C11—C12	117.48 (18)	O3—C19—H19C	109.5
C10—C11—H11	121.3	H19A—C19—H19C	109.5
C12—C11—H11	121.3	H19B—C19—H19C	109.5
C13—C12—C11	121.95 (19)	O4—C20—H20A	109.5
C13—C12—H12	119.0	O4—C20—H20B	109.5
C11—C12—H12	119.0	H20A—C20—H20B	109.5
C12—C13—C8	122.37 (19)	O4—C20—H20C	109.5
C12—C13—H13	118.8	H20A—C20—H20C	109.5
C8—C13—H13	118.8	H20B—C20—H20C	109.5
O5—Na1—O1	98.46 (6)	C20—O4—C21	114.7 (2)
O5—Na1—O4	71.18 (6)	C20—O4—Na1	130.10 (17)
O1—Na1—O4	105.07 (7)	C21—O4—Na1	109.70 (14)
O5—Na1—O2	167.64 (6)	O4—C21—C22	110.8 (2)
O1—Na1—O2	69.60 (6)	O4—C21—H21A	109.5
O4—Na1—O2	114.15 (7)	C22—C21—H21A	109.5
O5—Na1—O6	71.24 (6)	O4—C21—H21B	109.5
O1—Na1—O6	97.69 (6)	C22—C21—H21B	109.5
O4—Na1—O6	138.30 (6)	H21A—C21—H21B	108.1
O2—Na1—O6	106.35 (6)	O5—C22—C21	107.37 (18)
O5—Na1—O3	120.94 (6)	O5—C22—H22A	110.2
O1—Na1—O3	140.56 (6)	C21—C22—H22A	110.2
O4—Na1—O3	87.41 (6)	O5—C22—H22B	110.2
O2—Na1—O3	71.15 (6)	C21—C22—H22B	110.2
O6—Na1—O3	96.77 (6)	H22A—C22—H22B	108.5
O5—Na1—C16	144.27 (7)	C23—O5—C22	111.57 (15)
O1—Na1—C16	48.50 (7)	C23—O5—Na1	109.48 (11)
O4—Na1—C16	101.37 (7)	C22—O5—Na1	114.77 (12)
O2—Na1—C16	26.35 (6)	O5—C23—C24	107.43 (16)
O6—Na1—C16	119.74 (6)	O5—C23—Na1	45.17 (8)
O3—Na1—C16	92.73 (7)	C24—C23—Na1	78.98 (11)
O5—Na1—C23	25.36 (5)	O5—C23—H23A	110.2
O1—Na1—C23	110.27 (6)	C24—C23—H23A	110.2
O4—Na1—C23	90.17 (6)	Na1—C23—H23A	154.8
O2—Na1—C23	155.18 (6)	O5—C23—H23B	110.2
O6—Na1—C23	48.84 (5)	C24—C23—H23B	110.2
O3—Na1—C23	106.88 (6)	Na1—C23—H23B	88.8
C16—Na1—C23	157.75 (7)	H23A—C23—H23B	108.5
O1—C14—H14A	109.5	O6—C24—C23	108.80 (17)
O1—C14—H14B	109.5	O6—C24—H24A	109.9
H14A—C14—H14B	109.5	C23—C24—H24A	109.9
O1—C14—H14C	109.5	O6—C24—H24B	109.9
H14A—C14—H14C	109.5	C23—C24—H24B	109.9
H14B—C14—H14C	109.5	H24A—C24—H24B	108.3
C15—O1—C14	111.96 (18)	C24—O6—C25	111.52 (18)
C15—O1—Na1	115.51 (14)	C24—O6—Na1	112.19 (12)
C14—O1—Na1	122.87 (14)	C25—O6—Na1	120.14 (14)
O1—C15—C16	107.65 (18)	O6—C25—H25A	109.5
O1—C15—H15A	110.2	O6—C25—H25B	109.5
C16—C15—H15A	110.2	H25A—C25—H25B	109.5
O1—C15—H15B	110.2	O6—C25—H25C	109.5
C16—C15—H15B	110.2	H25A—C25—H25C	109.5
H15A—C15—H15B	108.5	H25B—C25—H25C	109.5
O2—C16—C15	107.43 (17)		
			
C8—C1—C2—C3	−161.55 (16)	C14—O1—C15—C16	−175.81 (19)
C1^i^—C1—C2—C3	17.1 (2)	Na1—O1—C15—C16	−28.6 (2)
C8—C1—C2—C7	21.4 (3)	O1—C15—C16—O2	59.8 (2)
C1^i^—C1—C2—C7	−159.90 (15)	O1—C15—C16—Na1	19.41 (14)
C7—C2—C3—C4	−2.6 (2)	C15—C16—O2—C17	177.87 (18)
C1—C2—C3—C4	−179.96 (17)	Na1—C16—O2—C17	−121.37 (18)
C2—C3—C4—C5	1.8 (3)	C15—C16—O2—Na1	−60.75 (18)
C3—C4—C5—C6	0.6 (3)	C16—O2—C17—C18	169.02 (18)
C4—C5—C6—C7	−2.1 (3)	Na1—O2—C17—C18	49.6 (2)
C5—C6—C7—C2	1.3 (3)	O2—C17—C18—O3	−64.0 (3)
C3—C2—C7—C6	1.1 (2)	C17—C18—O3—C19	−175.3 (2)
C1—C2—C7—C6	178.24 (17)	C17—C18—O3—Na1	44.6 (2)
C2—C1—C8—C9	−167.86 (16)	C20—O4—C21—C22	−108.3 (3)
C1^i^—C1—C8—C9	13.5 (2)	Na1—O4—C21—C22	48.4 (3)
C2—C1—C8—C13	15.4 (3)	O4—C21—C22—O5	−57.4 (3)
C1^i^—C1—C8—C13	−163.26 (15)	C21—C22—O5—C23	162.93 (18)
C1—C8—C9—C10	−179.18 (17)	C21—C22—O5—Na1	37.7 (2)
C13—C8—C9—C10	−2.1 (2)	C22—O5—C23—C24	178.34 (17)
C8—C9—C10—C11	0.7 (3)	Na1—O5—C23—C24	−53.51 (18)
C9—C10—C11—C12	1.1 (3)	C22—O5—C23—Na1	−128.15 (18)
C10—C11—C12—C13	−1.4 (3)	O5—C23—C24—O6	61.5 (2)
C11—C12—C13—C8	−0.1 (3)	Na1—C23—C24—O6	26.01 (14)
C1—C8—C13—C12	178.66 (17)	C23—C24—O6—C25	−175.77 (18)
C9—C8—C13—C12	1.8 (2)	C23—C24—O6—Na1	−37.7 (2)

Symmetry code: (i) −*x*+1, *y*, −*z*+1/2.
